# The effect on the patient flow in local health care services after closing a suburban primary care emergency department: a controlled longitudinal follow-up study

**DOI:** 10.1186/s13049-017-0460-3

**Published:** 2017-11-28

**Authors:** Katri Mustonen, Jarmo Kantonen, Timo Kauppila

**Affiliations:** 1 Department of Primary Health Care Laboratory Services, Helsinki University Central Hospital, Laboratory Services (HUSLAB), Topeliuksenkatu 32, 00029 HUS, Helsinki, Finland; 2 Primary Health Care, City of Vantaa, Peltolantie 2D, 01300 Vantaa, Finland; 3 Department of General Practice and Primary Health Care, Clinicum of Faculty of Medicine, University of Helsinki, (Tukholmankatu 8B), -00014 Helsinki, SF Finland

**Keywords:** Distance, Emergency department, Primary care, Suburban

## Abstract

**Background:**

It has not been studied what happens to patient flow to EDs and other parts of local health care system if distances to ED services are manipulated as a part of health policy in urban areas.

**Methods:**

The present work was an observational and quasi-experimental study with a control and it was based on before-after comparisons. The impact of terminating a geographically distant suburban primary care ED on patient flow to doctors in local public primary care EDs, office-hour primary care, secondary care EDs and in private primary care was studied. The effect of this intervention was compared with a primary care system where no similar intervention was performed. The number of monthly visits to doctors in different departments of health care was scored as the main measure of the study in each department studied (e.g. in primary care EDs, secondary care ED, office-hour public primary care and private primary care). Monthly mortality rates were also recorded.

**Results:**

Increasing the distance to ED services by terminating a peripheral ED did not cause an increase in the use of local office-hour services in those areas whose local ED was terminated, although use of ED services decreased by 25% in these areas (*P* < 0.001). The total use of primary care doctor services rather decreased - if anything - after this intervention while use of doctor services in secondary care ED remained unaffected. Doctor visits to the complementary private primary care increased but this was probably not associated with the intervention because a simultaneous increase in this parameter was observed in the control. There was no increased mortality in any age groups.

**Conclusion:**

Manipulating distances to ED services can be used to direct patient flows to different parts of the health care system. The correlation between distance to ED and the tendency to use ED by inhabitants is negative. If secondary care ED was available there were no life-threatening side-effects at the level of general public health when a minor ED was closed in a primary care ED system.

## Background

According to epidemiological studies, distance to an emergency department (ED) correlates negatively with the decision to use EDs [1–7]. When access is convenient, meaning the travel distance is short, patients are more likely to use an ED for less-urgent reasons [6, 7]. At the same time, various EDs suffer from overcrowding [8–10]. It has been suggested that this is due to inappropriate use of emergency services for health problems which do not require medical emergency actions [11–15]. Overcrowding is not an economic hazard if EDs are functioning under a pay-for-performance-system and non-public funding but it still compromises quality of work [10, 11]. This overcrowding causes considerable problems in non-profit-systems, such as the Finnish ED system. Unusually in an international context [16], it is divided into primary care and secondary care services and strongly based on general practitioners (GPs) [17, 18]. EDs and most of the office-hour primary care are funded by the public health system [17, 18]. To a small extent, primary care EDs are complemented by private primary care which is funded by patients’ own money and private insurance. Therefore, private primary care is not equally available to all Finnish citizens [19]. Both the public and the private sector primary care services, and the private secondary care service, consult the public secondary care service via referrals and the most difficult clinical cases are usually treated in the public secondary care service [17, 18]. In this publicly funded ED-system, overcrowding may therefore be the unwelcome side-effect produced by visits to doctors for less acute illnesses.

It has been postulated that, to ensure emergency treatment for those who need it most, distance factors should always be carefully considered when planning the location of an ED [1, 2]. However, the published research on the consequences of closing or restructuring primary care ED-services is scant. According to the only report which was found, increasing the distance to a semi-rural primary care ED-service by 40 km as the result of closing a local primary care ED, reduced overall use of primary care services [20]. The extent of this effect varied between genders [20]. No studies with control data was found. In 2005 Health authorities in Vantaa city also performed this type of intervention. They first noticed that of the two EDs in the city the smaller one, which was located 19 km away from the larger ED and performed the functions of a traditional Finnish primary care ED, treated mostly low acuity patients without need of immediate medical help. They closed this suburban ED in a geographically large city and centralized all ED functions in one large unit. Preliminary analysis of this intervention was published in Finnish in a doctoral dissertation [21] and therefore the experience gained from this study did not become well known. Concentrating ED services to less numerous but large units is right now a current trend in Finnish health care because of an ongoing social and health care reform (SOTE-uudistus). However, research about the putative effects of this activity is sparse.

The aim of the present experiment was to study how closing a geographically distant suburban ED alters patient flow to doctors in local public primary care EDs, office-hour primary care, secondary care EDs and, finally, in private primary care.

## Methods

### Setting

The present work is an observational and quasi-experimental study with a control and it was based on before-after comparisons. The intervention, namely the closure of a small suburban primary care ED, was performed in the city of Vantaa, which is the third largest city in Finland (roughly 182,000 inhabitants in 2005) and located just northeast of Helsinki. Vantaa is divided into five health care districts. The main primary care ED, Peijas, is located in Korso-Koivukylä district (“Control area A”, population about 46,000 inhabitants). In the eastern part of Vantaa city there are two other districts, Tikkurila (“Control area B”, the economic and administrative center of Vantaa city, about 47.000 inhabitants) and Hakunila-Länsimäki (“Control area C”, about 28.000 inhabitants). The two remaining health care areas are both located in the western part of Vantaa: the smaller primary care ED was located in Myyrmäki district (“Area X”, 34,000 inhabitants), and there is also the neighboring Martinlaakso district (“Area Y”, 26,000 inhabitants).

Because both primary and secondary care are provided in the ED at Peijas Hospital it is defined as a ‘combined ED’. It is equipped with out-of-hours laboratory and X-ray facilities, and primary care ED is carried out there only out of office hours. As a comparison, the primary care ED in “Area X” resembled a traditional Finnish primary health care out-of-hours unit, did not provide specialist care, and the laboratory and X-ray facilities were available only during office hours. This ED was not open during the night-time but only in the evenings and at weekends (for more detailed description see [17, 18]).

**Fig. 1:**
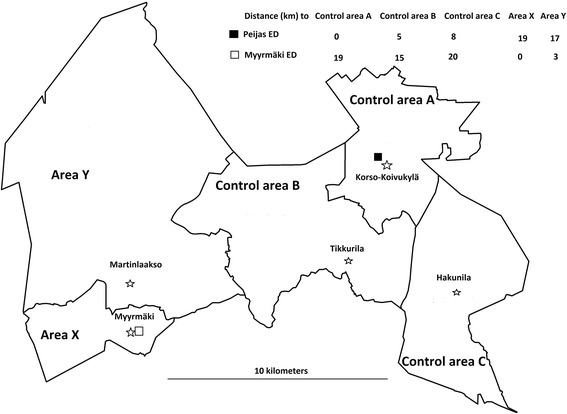
The map of Vantaa, its districts and EDs

Distances between districts were defined as point-to-point distances between the public primary care health centers which were without exceptions located in the economic, administrative and population centroids of the districts. The distances between the health care centers of these areas are presented in the Fig. 1. This measurement has been reported to correlate well with drive-times to the ED, which is the most accurate measurement for distance-related hindrances in access to an ED [6, 7] in similar type of studies, if there are no major geographical hindrances [22].

There is also a very similar city with about the same geographical size, population and ED system neighbouring Vantaa, namely, Espoo. Thus, it was possible to get control data for the intervention. At the time of the study this second largest city in Finland had a population of around 220,000 inhabitants and control data was analogously collected from the primary care EDs as we had done in our former work requiring control data for Vantaa [17]. The combined ED of Espoo was analogous to the combined ED of Peijas hospital in “Control area A” of Vantaa and it was located in Jorvi hospital. The other ED of Espoo in Puolarmetsä was similar to the primary care ED located in “Area X” of Vantaa.

To study the effect of closure of a small suburban ED on the total patient flows in EDs, the data obtained from Peijas ED in “Control area A” and the ED in “Area X” were pooled together as “Vantaa EDs’ data” and it was compared with “Espoo EDs’ data” obtained from both Jorvi and Puolarmetsä EDs. All the data were gathered and handled in such a way as to maintain patient and doctor anonymity. No ethical approval was required because this study was made directly by computer from the patient register without identifying the patients. The report generator automatically allowed following the monthly number of doctor visits in different departments of the local health system. The register keepers (the health authorities of Helsinki University Central Hospital [HUCH], Espoo and Vantaa and Social Insurance Institution of Finland [SII]) granted permission to carry out the study (23.8.2013).

### Main and secondary measures and data extraction

The number of monthly visits to doctors in different departments of health care was scored as the main measure of the study in each department studied (e.g. in primary care EDs, secondary care EDs, office-hour public primary care and private primary care). This was done before and after the closure of the ED in “Area X” (1.6. 2005). The data was obtained from the electronic health records of Vantaa (Finstar - patient chart system, Logica LTD, Helsinki, Finland) and Espoo primary health care (Effica- patient chart system, Tieto LTD, Helsinki, Finland) and Peijas and Jorvi secondary health care ED (HUCH; Musti and Oberon- patient chart systems). SII provided the data about the use of the private primary health care doctors. As a secondary outcome, monthly mortality rates were recorded (Finnish Statistics) in age groups 0–19, 20–64 and 65+ years to establish whether the present intervention represented any risk to general patient safety.

### Intervention

The intervention, namely the closure of a small suburban primary care ED in “Area X”, took place in the 1st June 2005. 2004 was the first year of the study because at the beginning of 2004 there was a major change in Vantaa primary care EDs when ABCDE-triage was applied [17]. Thus, in Vantaa the follow-up was performed from 1st February 2004 to 31st December 2007 after which Peijas ED moved to a new ED system (reverse triage) [23]. In Espoo (the control) the follow-up was performed from 1st February2004 to 1st April 2007, after which EDs moved gradually to a reverse triage system, which greatly altered patient flows in the local health care system [24]. Thus, we could study the situation before and after the intervention in the EDs of Vantaa and compare the changes with the situation in Espoo where no intervention was performed.

### Statistical methods

Both enumerative and statistical analytic methods were used [24]. Enumerative statistics were employed to determine whether the aggregated data from 2004, i.e. before intervention, differed significantly from the post-intervention situation. Since the “Area X” primary care ED closure took place at the beginning of May 2005, the number of patient visits before and after the closure were compared. The numbers of monthly visits to doctors were initially compared by using one-way repeated measures analysis of variance for abolishing the effects of systematic monthly variation caused by doctors’ holidays [24]. RM-Anova was followed by the Bonferroni’s correction.

The data were also evaluated by using analytic statistical methods (i.e., to look at data changes over time), with Statistical Process Control (SPC) tools (e.g. the XmR chart) [24–26]. Once the intervention (closure of the ED) was put in place, the performance of the dependent variable was compared to the baseline performance (February 2004 – May 2005). The SPC tests were used to determine if the process performance demonstrated common cause or special cause variation [25, 26]. Specifically, three statistical tests were applied to the data: a) A shift in the data demonstrated by 8 or more consecutive data points above or below the mean centreline on the control chart, b) A statistical trend in the data which is defined as 6 consecutive data points constantly increasing or decreasing, not counting values that are repeated in the sequence, and c) A data point that exceeds the upper (UCL) or lower (LCL) control limits on the control chart (i.e., a data point that exceeds 3 σ).

Pearson correlation coefficient was used to reveal putative correlation between distance to the ED and its use by calculating this coefficient between monthly patient visits to doctors of the nearest ED from different health care districts and the distance of these districts from this ED.

## Results

**Fig. 2:**
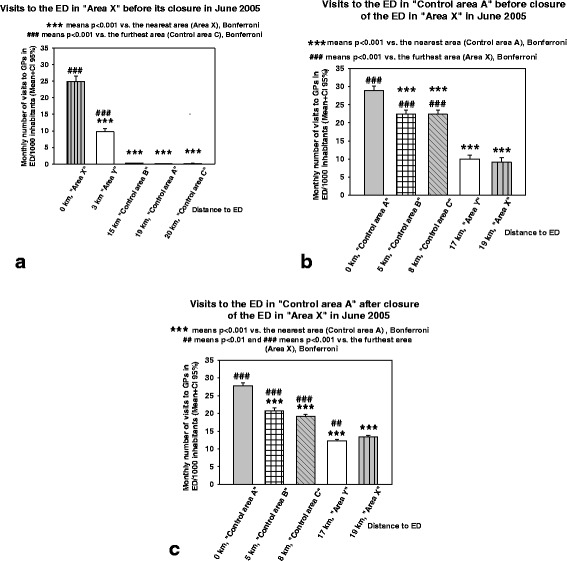
**a**) Number of monthly recorded patient visits to GPs as a function of distance to the ED in “Area X” before the closure of this ED. Mean is shown with bars and the size of 95% CI with a bracket. **b**) Number of monthly recorded patient visits to GPs as a function of distance to the ED in “Control area A” before the closure of the ED in “Area X”. **c**) Number of monthly recorded patient visits to GPs as a function of distance to the ED in “Control area A” after the closure of the ED in “Area X”

Before closing the “Area X” ED its use was most common among the inhabitants of “Area X” (RM-Anova; *P* < 0.001, Fig. 2a). It was also more frequently used by the inhabitants of “Area Y” than by the inhabitants of the remaining three control areas (Fig. 2a). During the same time-period, the Peijas ED was most used by the inhabitants of the nearest district, “Control area A” (*P* < 0.001), next by the inhabitants of the two next nearest districts, “Control area B” and “Control area C”, and, finally, least used by the inhabitants of the furthest two districts, “Area X” and “Area Y” (Fig. 2b). After the ED in “Area X” was closed and the Peijas ED became the only primary care ED serving the inhabitants of Vantaa, all the districts differed statistically significantly from each other (P < 0.001) in terms of monthly visits to primary care EDs’ doctors, so that the further the district was located from the ED, the fewer visits originated from that district (Fig. 2c). The only exception to this rule was that in the two furthest districts the number of visits to the doctors of the primary care ED was slightly lower in “Area Y”, whose population centroid was 2 km nearer to the Peijas ED, than that of “Area X”. There was a strong negative correlation (*r* = −0.876, *P* < 0.001) between distance of the health care district from the ED and the use of the EDs’ doctors by the inhabitants of these districts.

**Fig. 3:**
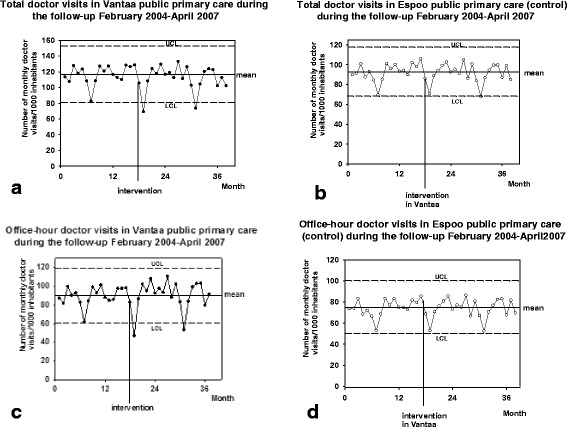
**a**) Total number of monthly recorded patient visits to GPs of Vantaa public primary care. The figure shows the original data in the form of an XmR-chart: mean ± 3 δ, e.g. UCL and LCL, are presented. **b**) Total number of monthly recorded patient visits to GPs of the control public primary care, Espoo. **c**) Number of monthly recorded office-hour patient visits to GPs of Vantaa primary care. **d**) Number of monthly recorded office-hour patient visits to GPs of the control primary care, Espoo

**Fig. 4:**
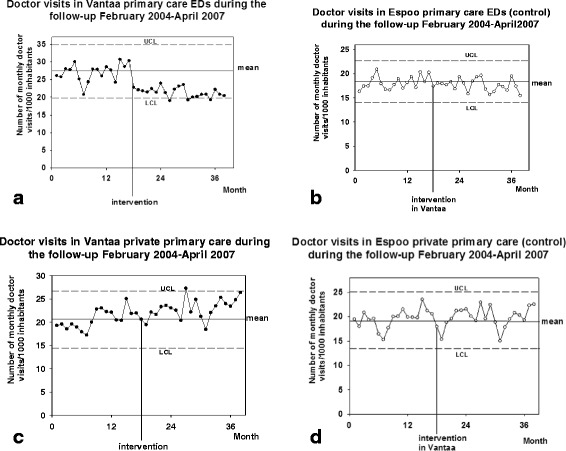
**a**) Number of monthly recorded patient visits to GPs of Vantaa primary care EDs. The figure shows the original data in the form of an XmR-chart: mean ± 3 δ, e.g. UCL and LCL, are presented. **b**) Number of monthly recorded patient visits to GPs of control primary care EDs in Espoo. **c**) Number of monthly recorded patient visits of inhabitants of Vantaa in private primary care doctors. **d**) Number of monthly recorded patient visits of inhabitants of Espoo, the control, in private primary care doctors

The total number of monthly visits to the doctors of Vantaa public primary care decreased (RM-Anova; *P* < 0.01) during the follow-up but this decrease was not temporally associated with the intervention (Fig. 3a). No change was observed in the control, e.g. public primary care of the control city Espoo (*P* = 0.252: Fig. 3b). There was no change observed in the visits to public primary care office-hour doctors in either of the cities (Vantaa; *P* = 0.116, and Espoo; *P* = 0.163: Fig. 3c, d). A decrease in monthly visits to the doctors of Vantaa public primary care ED-system (*P* < 0.001: Fig. 4a) was temporally associated with the intervention (Fig. 2a) but no similar changes were observed in the control city Espoo (*P* = 0.064: Fig. 4b). Visits to the private sector primary care doctors increased in the study population (P < 0.001), and among the inhabitants of the control city, Espoo (*P* < 0.05), where the number of monthly visits to private primary care GPs increased from 19.0 (17.8–20.1) in 2004 to 20.1 (18.8–21.4) in 2005 (Mean ± CI 95%, *P* < 0.01). This increase in the use of private primary care was neither clearly temporally associated with the intervention in Vantaa (Fig. 4c) nor in the control, Espoo (Fig. 4d).

**Fig. 5:**
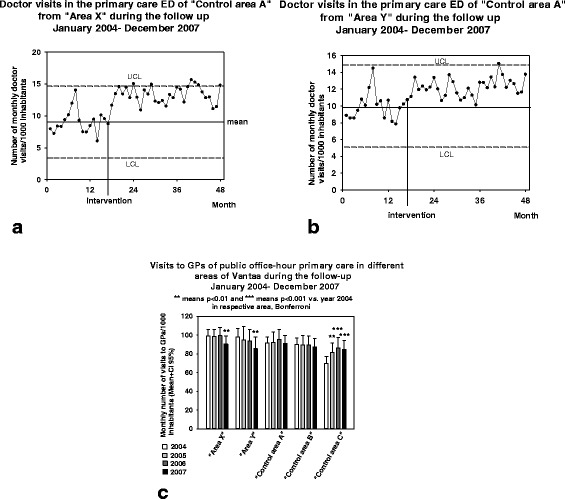
**a**) Number of monthly recorded patient visits from “Area X” to GPs of ED in “Control area A”. The figure shows the original data in the form of an XmR-chart: mean ± 3 δ, e.g. UCL and LCL, are presented. **b**) Number of monthly recorded patient visits from “Area Y” to GPs of ED in “Control area A”. **c**) Number of monthly recorded patient visits to the GPs of the office-hour primary care in different areas. Mean is shown with bars and the size of 95% CI with a bracket

The total number of monthly visits to GPs of the main primary care ED in “Control area A” was 21.5 (20.5–22.4) monthly visits/1000 inhabitants in 2004, 21.9 (20.0–22.8) in 2005, 21.0 (19.1–22.0) and 21.3 (20.4–22.2) showing no statistically significant changes (*P* = 0.243) during the follow-up. There was a marginal increase in the visits from those districts which were supplied by the closed primary care ED, e.g. “Area X” (*P* < 0.001) and “Area Y” (P < 0.001). These increases took place after terminating the “Area X” ED (Fig. 5a,b). In the public secondary care ED, there were 7.3 (7.0–7.6) monthly visits/1000 inhabitants (Mean ± CI 95%) to doctors in 2004, 7.4 (7.1–7.7) in 2005, 7.2 (6.9–7.5) in 2006 and 7.3 (7.0–7.6) in 2007, thus representing no statistically significant changes during the follow-up (*P* = 0.729). This was also the case with the Jorvi secondary care ED of the control city, Espoo (*P* = 0.074, detailed data not shown).

There was a decrease in the use of public primary care office-hour doctor services in those districts whose nearest ED was closed, i.e. in “Area X” (*P* < 0.01) and “Area Y” (P < 0.01). This decrease was not, however, temporally associated with the closure of “Area X” ED but took place in 2007 (Fig. 5c). Only in “Control area C”, which was thus 20 km away from the closed ED, an increase in monthly visits to the office-hour doctors (*P* < 0.001) was observed at the time of the intervention (Fig. 5c).

**Fig. 6:**
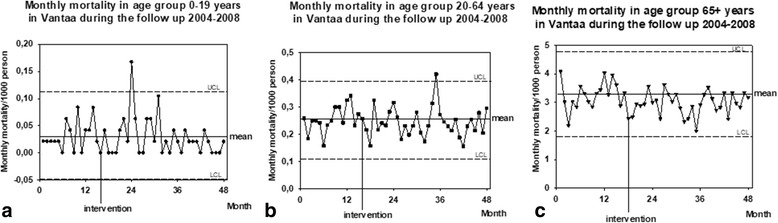
The monthly mortality in different age groups (**a** 0-19 years, **b** 20-64 years, and **c** 65+ years). The figures show the original data in the form of an XmR-chart: mean ± 3 δ, e.g. UCL and LCL, are presented

There was no increased mortality in any age groups (RM-Anova;*P* = 0.331 in 0–19 years; *P* = 0.512 in 20–64 years; *P* = 0.250 in 65+ years, Fig. 6).

## Discussion

Increasing the distance to ED-services of some inhabitants of a city by closing a peripheral ED decreased use of ED services in the suburbs located near the closed ED. This intervention did not cause an increase in the use of local office-hour services in those areas whose local ED was closed. The total use of the primary care doctor services rather decreased after this intervention while the use of doctor services in the secondary care ED remained unaffected. Doctor visits to the complementary private primary care increased but this was probably not associated with the intervention because a simultaneous increase in this parameter was observed in the control city.

Former epidemiological studies have suggested the existence of negative correlation between distance and the use of ED services [1–7]. In Norwegian primary care, the use of emergency primary care was reduced by approximately 1.5% per kilometre increased distance to the casualty clinic [27, 28]. Present data, with control data from a similar city without the intervention, provides additive experimental evidence [20] that there is a causal relationship between distance to ED and tendency to use ED by inhabitants.

We also confirm the results of Hansen et al. [20] suggesting that a decrease in ED services does not lead automatically to increase of office-hour services in other parts of health care. Those inhabitants who lost their nearest ED did not proceed instead to office-hour doctor services as one might have expected, but the use of these services rather decreased during the follow-up in the studied suburbs. The only increase we observed in the monthly visits to office-hour doctors took place in “Control area C” at the time of the intervention. There was no change in the ED supply of this suburb and the observed change had no direct connection to the present intervention because there was an increase in local office-hour doctor supply in public primary care just at the time of the observed change. Since there was also a simultaneous increase in the use of private primary care doctors in the control city, Espoo, the observed increase in this parameter in Vantaa was considered to reflect the general Finnish trend of the public increasingly using private sector primary care services [19].

“Selling inconvenience” by increasing traveling time to an ED [6, 7] was an effective way to decrease use of primary care ED services because there was a considerable decrease (about 5 visits/1000 inhabitants/month) in the use of the EDs’ doctors just after the present intervention. EDs may have “customers of their own” who do not, for various reasons, make use of other services [17, 29]. Epidemiological research from mixed urban and rural area suggests that the choice of type of unscheduled, out-of-hours health care may also be socially determined and that the effects of social deprivation may sometimes even overrun the effects of distance on care seeking behavior [30]. Interestingly, manipulating distances to EDs in the present situation did not lead to re-directing patients from EDs to more adequate office-hour primary care services. This re-directing is often suggested to be a method to decrease overcrowding in EDs [29, 31] and improve access to health services in less acute cases [32, 33]. This reluctance towards re-directing to office-hour primary care services can also be observed in a multicenter survey of patients from an urban health region. In this study, distance to a specific ED was the most important reason for choosing that service [34]. Nevertheless, our experimental data together with the former epidemiological [1–7, 27, 28], experimental [20] and survey studies [34] support the hypothesis that at least in urban areas manipulating distances to emergency services may be one tool to reduce use of EDs and thereby implement health policy [31, 35]. These results also suggest that there is a real causal relationship between the distance to the ED services and the use of these services. However, if closure of services is used as a tool in health policy, care should be taken that those areas which are socially deprived [33] are not located farthest from the remaining primary care and ED services.

There was no change in mortality which would have been temporally associated with the present intervention. Thus, there were no life-threatening side-effects at the level of public health when a minor ED was closed. Mortality, which has been used in similar types of studies as a definitive measure of safety in primary care interventions [36, 37] is not, however, a very sensitive indicator of safety.

### Limitations of the study

The Finnish ED system, based on GPs, may make the generalisation of the present results less applicable to secondary care driven EDs, which is the most commonly used type of ED system in other countries [16]. Secondly, the researchers were not consulted when the present intervention was planned. Therefore, other interventions in the ED system were started relatively soon after the present one and the follow-up period remained shorter than hoped. It is, unfortunately, very common in municipal interventions, that other interventions are applied even before the previous ones have been adequately evaluated. Furthermore, the researchers were not consulted regarding data collection.

Lack of data at individual patient level was a major shortcoming of the present study. With this type of data it would have been possible to determine exactly the distance the patients had to travel to reach the ED services. For example, having the possibility to use postcodes of the patients visiting the EDs [28] would have given us a lot of more information regarding the real travel distances to ED-services. Data at individual patient level would have provided more information on safety issues, too. Being able to follow individual patient cases would have offered the possibility to identify smaller negative impacts than deaths. Also, without data about individual patients, we cannot exclude the possibility that the patients were redistributed in such a way that was not the intention of the health care providers.

## Conclusions

At least in urban areas, manipulating distances to ED services can be used to direct patient flows to different parts of the health care system. The correlation between the distance to an ED and the tendency of inhabitants to use that ED is negative. The present data provides additional evidence for the hypothesis that there is also a causal relationship between distances to ED and the use of EDs.
